# Effects of Krill Oil on serum lipids of hyperlipidemic rats and human SW480 cells

**DOI:** 10.1186/1476-511X-7-30

**Published:** 2008-08-29

**Authors:** Jia-Jin Zhu, Jia-Hui Shi, Wen-Bin Qian, Zhen-Zhen Cai, Duo Li

**Affiliations:** 1 Department of Food Science and Nutrition, Zhejiang University, Hangzhou, PR China; 2 APCNS Centre of Nutrition and Food Safety, Hangzhou, PR China; 3 Department of Hematology, the First Affiliated Hospital, College of Medicine, Zhejiang University, Hangzhou 310003, PR China

## Abstract

**Background:**

Cardiovascular disease (CVD) and colon cancer incidence are known to be closely related to dietary factors. This article evaluated effects of krill oil (KO) on serum lipids of hyperlipidemia rats and human colon cancer cells (SW480). Serum lipids of rats fed with high fat diet (HFD) and different doses of KO were measured by automatic analyzer. Effect of KO on viability of cells was determined by methyl thiazolyl tetrazolium (MTT) assay.

**Results:**

Except for higher dose group, body weights decreased significantly. Total cholesterol (TC), LDL-cholesterol (LDL-C) of all dose groups, Triglycerides (TG) of low and mid dose groups descended significantly, while there were no significant differences of HDL-cholesterol (HDL-C), compared with control group. Treatment of colon cancer cells with KO also resulted in time-dependent inhibition of cell growth.

**Conclusion:**

Our findings indicated that the consumption of KO may provide benefits to control serum lipid levels in certain diseases and inhibit growth of colon cancer cells. Therefore, KO may be a good candidate for development as a functional food and nutraceutical.

## Background

Antarctic Krill are ancestors of clayfish and prawn. They have a slow evolutionary speed, and are not good at swimming. They are distributed in Vancouver saigan sea area, Russia, Ukraine and so on. They have the largest amount of protein among all organisms so far, over 16% in wet weight while over 65% in dry weight. Antarctic KO contain more than 30% of essential eicosapentaenoic acid (EPA, C:20:5, n-3) and docosahexaenoic acid (DHA, C:22:6, n-3) as well as astaxanthin (provitamin E) in concentrations of 200 – 400 ppm [1]. Besides, they also have abundant phospholipids, flavonoids, vitamin A, Alpha-linolenic acid (ALA), astacin and other nutrients [2].

Cardiovascular disease (CVD) and colon cancer incidence are known to be closely related to dietary factors [3]. As the modern medical science indicated, CVD had become the first killer of people's health. And diabetes, CVD and hypertension often cluster [4,5]. All kinds of dangerous factors, including change of life style, dietetic habit, indicate that the morbidity of coronary heart disease in China will rise quickly in the future. The latest survey of nutritional and health conditions of the citizens showed, prevalence of abnormal lipids among adults in China was 18.6%. Several recommendations based on experimental, epidemiological or nutritional data had shown that the incidence of CVD was positively correlated with saturated fatty acids intake and negatively with unsaturated fatty acids intake [6]. Controlled intervention trails with encapsulated fish oil supplements containing EPA plus DHA have established their triglyceride-lowering effects and modifying influence on other CVD risk factors independent of blood lipid-lowering [7,8]. Cancer chemoprevention has emerged as an important way to control cancer by using dietary agents capable of blocking neoplastic inception or delaying disease progression [9]. Research indicated that this strategy was promising on reducing the morbidity of cancer in high-risk population and in the common group [10]. In the recent years, the role of nutrients as chemopreventive agents has been the focus of current world. The idea of using natural extracts for the chemoprevention of cancer was obtained from animal models, clinical trials, human epidemiologic studies and cell studies [11]. Colon cancer is the second most common cause of cancer deaths in the USA, with an estimated annual incidence of 104,950 and mortality of 56,290 in 2005. The incidence of colon cancer is also constantly increasing in Asia [12]. A lot of effective methods and measures had been on service to reduce the mortality of colon cancer. Using naturally compounds present in dietary sources for chemoprevention is considered as a practical hopeful approach.

Some compounds have undergone clinical trials against colon cancer based on this idea [13,14]. The anti-cancer properties of KO are currently under investigation to see whether it can block or delay the malignant progression of transformed cells by modulating cell proliferation or differentiation. The cytostatic effect could be attained by the ingestion of KO on human colon carcinoma cell. Therefore, we studied whether KO had potential to slow the proliferation rates via programmed cell death. At present, studies are mainly focusing on the distribution, biological and physiological characteristics of Krill resources, few on the functional characteristics of KO. Therefore, the aim of this study was to evaluate the antilipidemic and anti-cancer effects of KO.

## Results

### Establishment of hyperlipidemia rats model

The results showed that the total TC, TG, and LDL-C of serum of rats significantly increased (P < 0.05) after 2 weeks of HFD, while high HDL-C decreased significantly (P < 0.05) compared with pre-feeding. It indicated the hyperlipemia models were established successfully. Thus, the following experiment was feasible.

### Effects of KO on body weights

**Figure 1:**
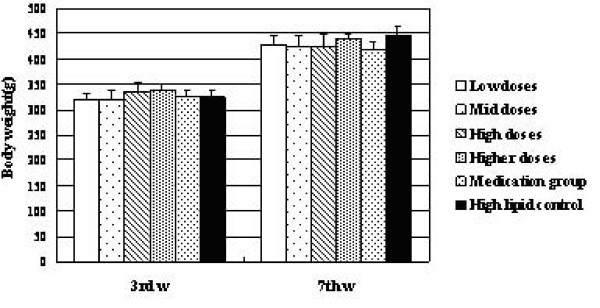
**Effects of KO on body weights of rats**. The body weight of experimental rats ($\bar{\text{x}}$ ± s, g, n = 10) in the third (3rd w) and seventh (7th w) weeks. *, p < 0.05, compared with high lipid model control group; **, p < 0.01, compared with high lipid control group; a, p < 0.05, compared with medication group.

Our study showed that, there were no significant differences, in body weight levels in other groups compared with the control group before administration (Figure 1). On the other hand, after four weeks' treatment with KO and lovastatin, diversity was observed. Except for Higher dose group, the body weight was decreased in each group obviously compared with control (p < 0.05). Among them, the difference between medication and control group was up to super remarkable level (p < 0.01). In addition, compared with medication group, there were no diversities in low, mid and high dose groups.

### Effects on the lipid levels

**Figure 2:**
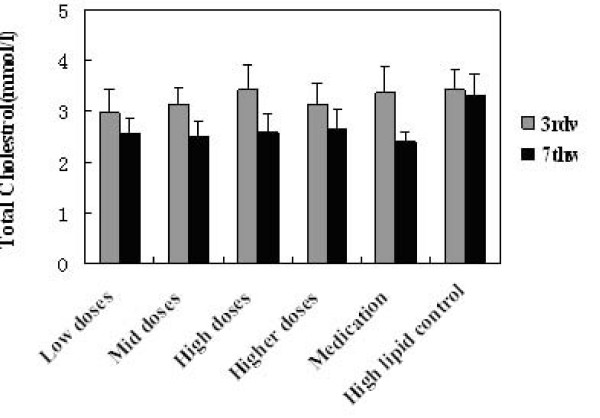
**Effects of KO on Total Cholesterol content**. Serum Lipids levels obtained after treatments with KO and lovastatin. The rats were fed for 4 weeks continuously. Data were mean values ± std for 10 rats.

**Figure 3:**
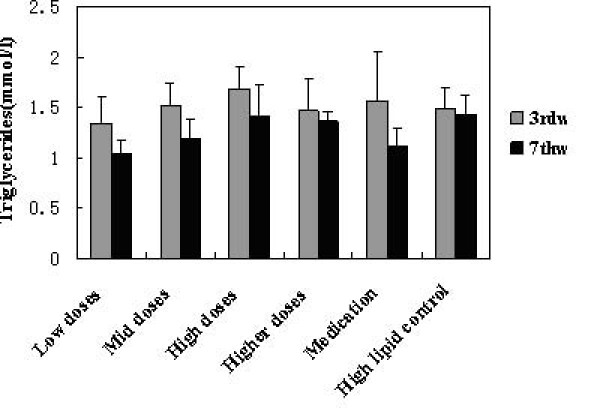
**Effects of KO on Triglycerides content**. Serum Lipids levels obtained after treatments with KO and lovastatin. The rats were fed for 4 weeks continuously. Data were mean values ± std for 10 rats.

**Figure 4:**
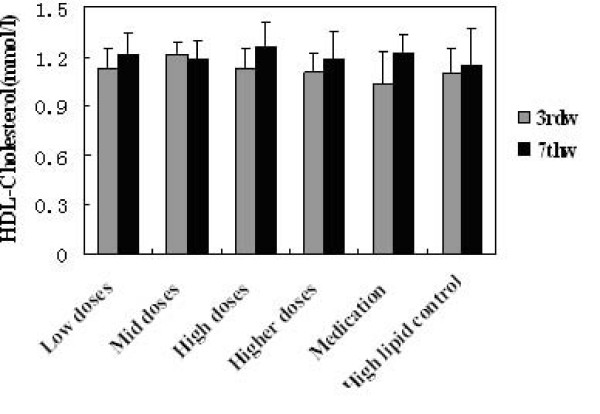
**Effects of KO on HDL-Cholesterol content**. Serum Lipids levels obtained after treatments with KO and lovastatin. The rats were fed for 4 weeks continuously. Data were mean values ± std for 10 rats.

**Figure 5:**
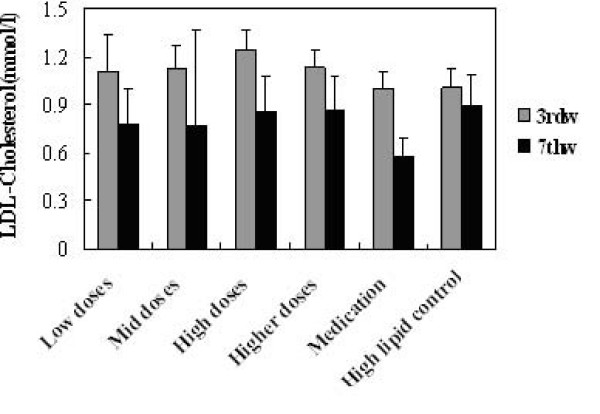
**Effects of KO on LDL-Cholesterol content**. Serum Lipids levels obtained after treatments with KO and lovastatin. The rats were fed for 4 weeks continuously. Data were mean values ± std for 10 rats.

TG, TC, HDL-C and LDL-C levels in serum before and after administration of KO were analysed. Significant changes in serum lipids were observed after 4 week consumption of KO. The intake of KO for 4 weeks had a lowering effect on TG, TC and LDL-C, while no changes were observed in HDL-cholesterol. All doses of KO could significantly decrease the TC (Figure 2), TG (Figure 3) and LDL-C (Figure 5) level of serum (P < 0.05) after 4 weeks of KO; high and higher doses of KO could significantly increase the HDL-C (Figure 4) levels. And compared with the hyperlipidemia control group, TC and LDL-C of all dose groups, TG of low and mid dose groups decreased significantly (p < 0.05), and no remarkable differences were observed. It indicated that KO could decrease TC, TG and LDL-C levels of serum significantly. On the other hand, it had little influence on serum HDL-C.

### Anti-cancer effect

**Figure 6:**
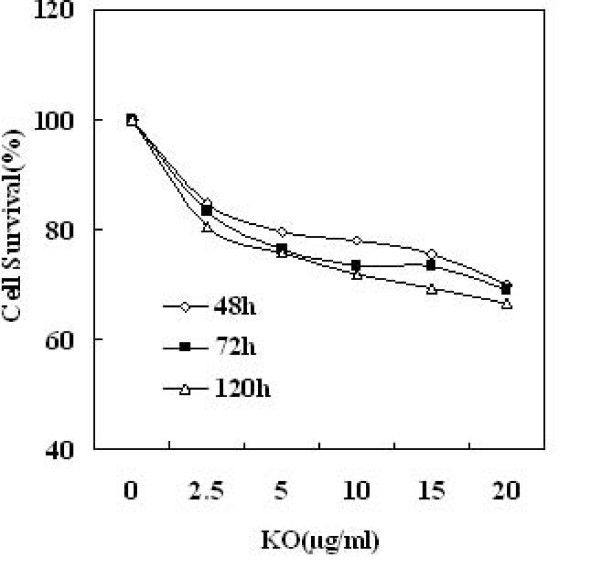
**Effect of KO on cell viability time-dependent in human colon cancer SW480 cells**. The cells were exposed to the specified concentration of KO for 48 h, 72 h and 120 h, and viability of cells were determined by MTT assay. Cell viabilities were described as percentages; vehicle-treated cells were regarded as 100% viable. Details were described in methods.

As was shown in Figure 6, treatment of SW480 cells with 2.5, 5, 10, 15, 20 μg/ml concentrations of KO for 48 h, resulted in 15.2%, 20.6%, 22.0%, 24.6% and 29.9% inhibition of cell growth respectively, compared with vehicle treated control. Exposure of SW480 cells to KO resulted in slight decrease in cell viability at the highest concentration of 50 μg/ml (data not shown). Treatment of colon cancer cells with KO also resulted in time-dependent inhibition of cell growth, and the effect was more pronounced at 72 h post-treatment. The inhibition rates were 16.7%, 23.4%, 26.7%, 27.2% and 30.9%, respectively. While in 120 h, the numbers were 19.7%, 24.2%, 28.0%, 30.8% and 33.5%, respectively.

## Discussion

It suggested that administration of KO caused significant change in body weight and the KO had good weight lose effect similar to the lipid-reducing medicine. It has also been reported that different types of fatty acids have different effects on body weight gain and insulin resistance. Saturated fatty acids (SFAs) produce more weight gain and insulin resistance than polyunsaturated fatty acids (PUFAs) in some studies [15,16]. Of all the PUFAs, EPA and DHA exert more favorable influence on body weight [17]. It was reported body weight of the diet group supplement with fish oil increased by 25% [18]. Fatty acids derived from fish oil had been shown to alter proinflammatory cytokine production and acute-phase protein (APP) synthesis in vitro. The presence of APP has been suggested to contribute to weight loss. The administration of PUFAs to hepatocytes had suggested that EPA may have direct effects on the modulation of APP production [19]. The mechanism of the KO used in the present study and the relative contributions of its components requires further study.

The present study proved that KO could significantly decrease the TC, TG and LDL-C levels of serum, while slightly increase the HDL-C levels as well. It was reported the long-term intake of egg-white hydrolysed (hEW) for 20 weeks had a lowering effect on TG and TC, while no changes were observed in HDL-C [20]. It was also found that some natural active substances could significantly decrease the TC, TG and LDL-C levels, but there were no significance in HDL-C [21,22]. A great deal of findings indicated that EPA and DHA could reduce body blood TG, TC and LDL-C. It had been reported that fish oil had cholesterol-lowering effect because it increased DHA content in the membrane and could improve membrane fluidity. Thus, it increased removal rate of VLDL-C and LDL-C particles from plasma by improving hepatic microsomal membrane fluidity, and thus, it decreased the TG, TC, LDL-C significantly. But it didn't increase the HDL-C level. Unlike fish oil and KO, corn oil significantly increased plasma HDL-C levels and reduced the risk factor for CVD since HDL-C had the important role of reversing cholesterol transport [23]. The reason may be the difference of various food composition, operation mechanism, or different animal study itself (great individual differences), the complex of metabolism of HDL-C and so on.

As far as we know, this is the first report showing the anti-cancer effect of KO on human colon cancer cells. However, more detailed studies are required to determine the exact mechanism(s). KO contains four constituents which may reduce the risk of developing the colon cancer: EPA, DHA, ALA, sphingolipid. Many reports indicated that EPA and DHA could inhibit the growth of some cancer cells, such as breast, prostate cancer [24,25]and so on. Fish oil was known to reduce growth of certain tumors, such as colon, breast and prostate cancers [26]. These effects were attributed to the content of PUFAs of the n-3 family in fish oil, in particular EPA and DHA, which regulate cellular signaling paths. Several PUFAs are known regulators of the ligand-activated transcription factors known as peroxisome proliferators-activated receptors (PPARs) [27]. Originally implicated in the regulation of lipid metabolism and adipocyte differentiation, the PPARs have also been implicated in cell differentiation, cell proliferation, and in inflammatory responses [5,25]. However, the mechanism(s) underlying of the anti-cancer effects were not fully understood. ALA alters the fatty acid composition of cell membranes in crucial ways and inhibits the release of pro-inflammatory eicosanoids, which control the growth and invasiveness of tumor cells and modulate the cycle of cell apoptosis among the many factors [28].

As was known, sphingomyelin which existed in cell plasma membrane of most mammalian was the main component of myelin sheath. It was rich in cell membrane of brain and nerve. Several studies had demonstrated altered total sphingolipid composition, both increases and decreases, in cancer cells. It is unclear what effect changes in sphingolipid composition and metabolism(s) have on the sensitivity of cancer cells to therapy. It was reported that dietary sphingomyelin protected against apoptosis and hyperproliferation caused by the hydrophobic bile salt deoxycholate potential implications for colon cancer. They thought the use of sphingomyelin to boost the chemotherapy response of cancer cells could have a significant impact on treatment outcome [29]. Numerous epidemiologic studies have provided a gist for the development cancer chemoprevention protocols using bio-active dietary agents capable of eliminating pre-malignant or malignant cells. The results of this study suggested KO had the potential to affect the steady state cell population.

## Conclusion

The main finding of this study was that the treatment with KO reduced the body weight and serum TG, TC and LDL-C levels significantly. The results showed hypolipidemic properties and thus, the consumption of KO may provide benefit to control serum lipid levels in certain diseases. In addition, KO affected the steady state cell population to inhibit growth of colon cancer and thus may be a good candidate for development as a chemopreventive and/or therapeutic agent against colon cancer. In a word, it is hopeful for KO to be one kind of potential functional food.

## Methods

### Preparation of KO

The krill oil was purchased from local market. The oil was placed at the refrigerator with the temperature of -20°C. Distilled water was added to Ryoto sugar ester (S-1170F, Mitsubishi-Kagaku Foods Corporation) to make the concentration 5 g/L, and then the solution was mixed for 10 minutes at the speed of 1500 rpm using magnetic whisk mix. The final solution was used as the solvent for KO to make desired samples for rats.

### Cell lines

Human colon cancer cells, SW480 were purchased from Stem Cell Bank, Chinese Academy of Sciences, and were propagated in the recommended medium. And the cells were cultured in RPMI 1640 medium with 10% FBS and 1% penicillin streptomycin (GIBCO) at 37°C in a incubator of humidified atmosphere with 5% CO_2_.

### Animal study

Sixty adult male SD rats, weighing 180 ~ 190 gram each, were purchased from Zhejiang Academy of Medical Science, and divided into six groups. Ten rats of each group were housed in one cage and fed in the center for experimental animals of Zhejiang University with the room temperature (25 ± 2)°C and humidity (63 ± 2)%. The HFD was composed of 78.8% common feed, 1% cholesterol, 10% yolk powder, 10% lard and 0.2% cholate. Body weights and feed intakes were recorded every three days during the next seven weeks. Following was the animal study procedure: SD rats were kept in SPF animal experiment lab for 1 week with fundament diet. Serum samples were taken from rats' tails and centrifuged at 3000 rpm for 15 min at 4°C for the analysis of TC, TG, HDL-C and LDL-C levels. Then rats were fed with HFD for 2 weeks to establish the hyperlipidemia model according to TC, TG, HDL-C and LDL-C levels. And then the rats were divided into 6 groups according to the TC levels randomly. The low dose group was given HFD with 16.65 g/L of KO, and the mid dose, the high dose and the higher dose groups were feed by HFD supplemented with 33.3 g/L, 99.9 g/L and 199.8 g/L of KO, respectively. The high lipid control was given HFD with the same volume solvent. The dosages of KO were 0.5 ml/100 g body weight of experimental rats. And the medication group was treated with lovastatin 100 mg/kg/day. Serum from 12 h-fasting rats' tails was taken after 1 w, 3 w and 7 w. The analysis of TC, TG, HDL-C and LDL-C were measured by automatic hitachi-7170 analyzer.

### Cell proliferation assay

The effect of KO on the viability of cells was determined by methyl thiazolyl tetrazolium (MTT) (3-[4,5-dimethylthiazol-2-yl]-2,5-diphenyl tetra zoliumbromide) assay. In short, the cells were plated at 1 × 104 cells per well in 200 μl of complete culture medium containing 0, 2.5, 5, 10, 15, and 20 μg/ml concentrations of KO in 96-well microtiter plates. The KO solutions were dissolved in anhydrous ethanol and mixed with aseptic PBS to achieve the desired final concentration. Each concentration of KO was repeated in ten wells. After incubation at 48 h, 72 h, 120 h in the incubator, cell viability was determined. Twenty microlitres MTT (5 mg/ml in phosphate-buffered saline stock, diluted to working strength 1 mg/ml with media) was added to each well and incubated for 4 h. After that, the MTT solution was removed from the wells by careful aspiration. Then, 200 μl of buffered DMSO was added to each well and plates were well-mixed. The absorbance was recorded on a microplate reader at the wavelength of 490 nm. The effect of KO on growth inhibition was evaluated as percent cells viability where vehicle KO-treated cells were considered as 100% survival.

### Statistical analysis

All data were handled with SPSS package program version 11.0 (SPSS, Chicago, IL). The results were listed in the way of mean ± std ($\bar{\text{x}}$ ± s), and the significance between the control and treated groups was performed using ANOVA Statistical analysis. Differences were considered significant at P < .05.

## List of abbreviations used

ALA: alpha-linolenic acid; CVD: cardiovascular disease; DHA: docosahexaenoic acid; EPA: essential eicosapentaenoic acid; HFD: high fat diet; KO: krill oil; MTT: methyl thiazolyl tetrazolium; PPARs: peroxisome proliferators-activated receptors.

## Competing interests

The authors declare that they have no competing interests.

## Authors' contributions

JJZ participated in the design of the study and carried out the statistical analysis. JHS performed the animal and cell studies, participated in preparing the test materials and writing of the manuscript. WBQ participated in the design of the cell study. ZZC participated in the revision of the manuscript. DL conceived of the study, and took part in its design and coordination. All authors read and approved the final manuscript.

## References

[B1] Kolakowska A, Kolakowski E, Szczygielski M (1994). Winter season krill (Euphausia superba Dana) as a source of n-3 polyunsaturated fatty acids. Die Nahrung.

[B2] Ruben B, Luis R, Katy Y, Georgina D, Claudio R (2003). Oxidative stability of carotenoid pigments and polyunsaturated fatty acids in microparticulate diets containing krill oil for nutrition of marine fish larvae. J Food Eng.

[B3] Haenszel W, Kurihara M (1968). Studies of Japanese migrants. I: Mortality from cancer and other diseases among Japanese in the United States. J Natl Cancer Inst.

[B4] Tan CE, Emmanuel SC, Tan BY, Tai ES, Chew SK (2001). Diabetes mellitus abolishes ethnic differences in cardiovascular risk factors: lessons from a multi-ethnic population. Atherosclerosis.

[B5] Grimaldi PA (2001). Fatty acid regulation of gene expression. Curr Opin Clin Nutr.

[B6] Trautwein EA, Rieckhoff D, Kunath-Rau A, Erbersdobler HF (1999). Replacing saturated fat with PUFA-rich (sunflower oil) or MUFA-rich (rapeseed, olive and high-oleic sunflower oil) fats resulted in comparable hypocholesterolemic effects in cholesterol-fed hamsters. Ann Nutr Metab.

[B7] Harris WS (1996). N-3 fatty acids and lipoproteins: comparison of results from human and animal studies. Lipids.

[B8] Harper CR, Jacobson TA (2005). Usefulness of omega-3 fatty acids in the prevention of coronary heart disease. Am J Cardio.

[B9] Tsao AS, Kim ES, Hong WK (2004). Chemoprevention of cancer. Cancer J for Clinicians.

[B10] Edwards BK, Brown ML, Wingo PA, Howe HL, Ward E, Ries LA, Schrag D, Jamison PM, Jemal A, Wu CX, Feiedman C, Harlan L, Warren J, Anderson RN, Pickle LW (2005). Annual report to the nation on the status of cancer, featuring population-based trends in cancer treatment. J Natl Cancer Inst.

[B11] Mehta RG, Pezzuto JM (2002). Discovery of cancer preventive agent from natural products: from plants to prevention. Curr Oncol Rep.

[B12] Jemal A, Murray T, Ward E, Samuels A, Tiwari RC, Ghafoor A, Feuer EJ, Thun MJ (2005). Cancer statistics. Cancer J for Clinicians.

[B13] Boone CW, Kelloff GJ, Malone WE (1990). Identification of candidate cancer chemopreventive agents and their evaluation in animal models and human clinical trials: a review. Cancer Res.

[B14] Reddy BS (2000). Novel approaches to the prevention of colon cancer by nutritional manipulation and chemoprevention. Cancer Epidemiol Biomarkers Prev.

[B15] Larson DE, Hunter GR, Williams M, Kekes-Szabo T, Nyikos I, Goran MI (1996). Dietary fat in relation to body fat and intraabdominal adipose tissue: A cross-sectional analysis. Am J Clin Nutr.

[B16] Loh MY, Flatt WD, Martin RJ, Hausman DB (1998). Dietary fat type and level influence adiposity development in obese but not lean Zucker rats. Proc Soc Exp Biol Med.

[B17] Storlien LH, Jenkins AB, Chisholm DJ, Pascoe WS, Khouri S, Karegen EW (1991). Influence of dietary fat composition on development of insulin resistance in rats. Relationship to muscle triglyceride and n-3 fatty acids in muscle phospholipids. Diabetes.

[B18] Dieter VA, Marian B, Heinz R, Ruthard J (1988). Influence of a diet rich in fish oil on blood pressure, body weight and cardiac hypertrophy in spontaneously hypertensive rats. Eur J Appl Physiol.

[B19] Wigmore SJ, Fearon KCH, Ross JA (1997). Modulation of human hepatocyte acute phase protein production in vitro by n-3 and n-6 polyunsaturated fatty acids. Ann Surg.

[B20] María AM, Marta M, Jeanne E, Rosario H, Amaya A, Rosina LF (2007). Effect of the long-term intake of an egg white hydrolysate on the oxidative status and blood lipid profile of spontaneously hypertensive rats. Food Chem.

[B21] Zhao P, Li B, Yang JF, Liu RZ, He WT, Li F (2005). Effect of Natural Taurine on Reducing Blood Lipids. Acta Nutrimenta Sinica.

[B22] Yang XQ, Zhang HD, Li L (2004). The Antioxidants and Antilipemic effects of Flavonoids extracted from Pomelo Peel. Acta Nutrimenta Sinica.

[B23] Park HS, Choi JS, Kim KH (2000). Docosahexaenoic acid-rich fish oil and pectin have a hypolipidemic effect, but pectin increases risk factor for colon cancer in rats. Nutr Res.

[B24] Maillard V, Bougnoux P, Ferrari P, Jourdan ML, Pinault M, Lavillonniere F, Body G, Le Floch O, Chajès V (2002). N-3 and n-6 fatty acidsin breast adipose tissue and relative risk of breast cancer in a case-control study in Tours, France. Int J Cancer.

[B25] Norrish AE, Skeaff CM, Arribas GLB, Sharpe SJ, Jackson RT (1999). Prostate cancer risk and consumption of fish oils: a dietary biomarker-based case-control study. Brit J Cancer.

[B26] Terry PD, Terry JB, Rohan TE (2004). Long-chain (n-3) fatty acid intake and risk of cancers of the breast and the prostate: recent epidemiological studies, biological mechanisms, and directions for future research. J Nutr.

[B27] Jump DB (2002). The biochemistry of n-3 polyunsaturated fatty acids. J Biol Chem.

[B28] Zhou JR, Blackburn GL (1997). Bridging animal and human studies: what are the missing segments in dietary fat and prostate cancer?. Am J Clinl Nutr.

[B29] Moschetta A, Portincasa P, van Erpecum KJ, Debellis L, vanBerge-Henegouwen GP, Palasciano G (2002). Dietary sphingomyelin protects against apoptosis and hyperproliferation induced by the hydrophobic bile salt deoxycholate potential implications for colon cancer. Digest Liver Dis.

